# The emergence of new obsessions and compulsions after COVID-19: a case report

**DOI:** 10.1192/j.eurpsy.2024.1049

**Published:** 2024-08-27

**Authors:** M. Abdelkefi, R. Feki, I. Gassara, N. Smaoui, S. Omri, N. Charfi, L. Zouari, J. Ben Thabet, M. Maalej Bouali, M. Maalej

**Affiliations:** Psychiatry C department, Hedi Chaker university hospital, sfax, Tunisia

## Abstract

**Introduction:**

The coronavirus pandemic has affected mental health since its outbreak in 2019 and several studies have revealed that obsessive-compulsive disorder (OCD) patients were adversely affected.

**Objectives:**

The aim of our present report is to study the impact of the coronavirus infection on OCD.

**Methods:**

We illustrate a case of new emerging obsessions and compulsions after a COVID-19 infection in a patient consulting at the Psychiatry C department of the Hedi Chaker University Hospital.

**Results:**

Mr. HB first presented in our outpatient unit of the Psychiatry C department at the age of 32. His medical history was unremarkable except for a COVID-19 infection in June 2022 that did not cause any organic complications. His family history was negative without neurological and psychiatric diseases. Further history revealed that OCD symptoms already started before his infection with COVID-19. At that time, he suffered from compulsions with the urge to constantly wash his hands and check rituals, but his symptoms were not severe enough to make him seek a psychiatric consult or treatment. However, since July 2022, soon after his infection with COVID-19, he suffered from new obsessions and compulsions, he would spend hours calculating all the numbers he sees and counting the number of letters in the words he came across. Non-surprisingly, OCD symptoms caused relevant problems in social life, and at work, he had difficulties concentrating and working. Due to his symptoms, he had neglected hobbies, avoided social contact, spent less time with his family, and even had suicidal thoughts. Therefore, pharmacotherapy with clomipramine was initiated with a maximal dose of 150 mg/d. Because of adverse events and lack of efficacy, he stopped medication and decided to consult our department to seek a different treatment. Therefore, sertraline was started at the dose of 50 mg/d along with psychotherapy.

**Conclusions:**

People with OCD are likely to be more susceptible to the mental health impact of COVID-19. Responses to the pandemic are not only associated with an increase in all Obsessive-Compulsive (OC) symptoms and their severity but also the emergence of new types of obsessions and compulsions. Consequently, therapists should consider the effects of the pandemic on all OC symptom dimensions and adjust their treatment plans accordingly.

**Disclosure of Interest:**

None Declared

